# LinkAGES Colorado: Evaluation of Intergenerational Programs to Improve Connections Across All Ages

**DOI:** 10.1093/geroni/igab046.2290

**Published:** 2021-12-17

**Authors:** Carson De Fries, Leslie Hasche, Rachel Cohen, Andrew Steward, Matthew Schilz

**Affiliations:** 1 University of Denver, Denver, Colorado, United States; 2 Aging Dynamics, Pine, Colorado, United States; 3 University of Denver, Lone Tree, Colorado, United States

## Abstract

LinkAGES: Colorado is a collaborative group of multi-sector organizations (e.g., libraries, non-profit service organizations, nursing homes) that uses a capacity-building approach to support the offering of intergenerational programs and evaluation of outcomes over time and across programs. Since 2018, LinkAGES has implemented 20 intergenerational programs involving various activities (e.g., music and art therapy, sharing cultural traditions), across settings, and across modalities (in-person and online). Ages of program participants ranged from 5 months to 96 years old. This study evaluated change in connectedness between generations over time. Participants (n=118) completed pre- and post-program ratings on social connection (i.e., level of intergenerational engagement, self-perception of extent of feeling connected, and self-perception of impact on someone from another generation) on a 4-point Likert scale. Paired sample t-test results indicated that programs significantly improved engagement and perceived impact. Using multiple regression analyses, we tested change over time for each outcome controlling for participant age group, program host setting, and program modality. A greater positive change in level of engagement occurred for older adults and in-person programs. Additionally, feelings of connection and perceived impact significantly improved over time when controlling for age group, program modality, and program host setting, with age group as a significant covariate. This study demonstrates the impact of intergenerational programs on social connectedness across a wider network of organizations than much of the extant literature. While the positive outcomes are promising and consistent across LinkAGES programs and existing literature, further exploration of age group differences should be considered.

